# Sanitary Commission of New Orleans

**Published:** 1857-03

**Authors:** 


*Sanitary Commission of New Orleans of* 1853.—*Public Appreciation of Scientific Labors.*—Every medical man has heard of the "Report of the Sanitary Commission of New Orleans, on the Epidemic Yellow Fever of 1853, published by authority of the City Council of New Orleans," a voluminous volume, the greater part of which was the production of the pen of Dr. E. H. Barton, and a work, too, which has elicited much remark as well as provoked not a little criticism. The history of the organization of this commission is easily told. The great epidemic of 1853 startled the public from the sense of false security in which they had been guilty reposing, and induced a determination to look into the subject, and to ascertain, if possible, if all this misery and loss of life was inevitable, or whether the hand of man did not give additional vigor to the arrows of disease and death which the skeleton arm of the grim archer held ready poised, but might hesitate to hurl.

The Board of Health appointed a commission, consisting of the Honorable Mayor of the city, and five medical men. To this commission was assigned the duty of making investigations into the origin and mode of transmission or propagation of the late epidemic yellow fever, and to make a thorough examination into the sanitary condition of the city, with the collateral subjects of sewerage, drainage, and quarantines.

The commission immediately organized and proceeded to the fulfillment of the important task assigned to it. From the report, we learn it issued circulars embracing all the points suggested for examination, and distributed them among the medical faculty and citizens in every quarter of the yellow fever region, whence information could be expected on the subjects under consideration. It sat as a court of inquiry, in that city, daily, for about three months, eliciting and inviting information from every accessible source. It then deputed its various members to visit different parts of their own and the adjoining states, to institute inquiries upon the subjects before them; one member visited the eastern states; and the aid of the general government, at Washington, was invoked to assist them in obtaining information abroad, throughout the yellow fever zone, to throw light upon the subjects of their study.

The results of these labors was given to the public in a volume of some five hundred and fifty pages. The report is a monument to the industry and zeal of the commission, but it told a tale which could not have been very flattering to the pride of their citizens, as it recorded the defects of their past municipo-sanitarial condition and government. But as we read upon its title, "Published by authority of the City Council," it seemed to us as the voice of repentance over past errors, and the promise of future reformation.

We have frequently, as we have turned the pages of this report, with its numerous maps and tables, the price of the toil of so many hours of Dr. Barton and his associates, wondered at and admired the liberality of that municipal government which had designed and executed so elaborate a sanitary work. We have wondered, too, if even the yellow fever would enlarge the hearts and expand the pockets of our northern city-fathers, and elicit such an acknowledgment of the value of medical labors, and by at least one act of liberality make an atonement for numberless wrongs done to the medical profession; for we had never the least doubt, but that for all this toil New Orleans had remembered to pay the commissioners well for their labors.

But alas! our faith was too great, and we have to mourn over the renewed evidence of the ungratefulness of the public, and the uncertainty which ever attends the collection of the bill of the medical man; for New Orleans has not paid the commissioners for their labor, and they have been compelled to bring a suit to obtain a just recompense for their services performed in behalf of the city's welfare! The commission is now searching over the length and breadth of our land, for testimony to establish before a court "the value of the services rendered," as every medical man is compelled to do who has the temerity to ask the aid of the law in obtaining his just dues; as if labors such as those performed by this body, could be measured by dollars and cents, and rendered tangible to the dull senses of the pennywise, as is the per centage of their stock investments, and the interest upon their bonds and mortgages. Certainly the laborer is worthy of his hire, and it is difficult to conceive of a reasonable basis of opposition to the claims they make for the value of their time and services rendered.

Years have elapsed since the visitation of the epidemic; the dead are burried out of sight, and forgotten; the pestilence has hidden itself in the depths of the swamps and the sloughs of the town; the black wing of the angel of death has been folded; security has taken the place of alarm; the dance of life goes joyously on; and the men who did not only sentry duty upon the very outposts of danger, but as scouts, penetrated into the heart of the enemy's camp, and sought to disarm him there, are forgotten in the hour of safety, and when life's channels are again filled by the returning waves of the full tide of business and commercial prosperity!

We know enough of the defence, to infer that an effort will be made to establish the point, that the deductions of the commission are purely hypothetical, and that we yet remain in ignorance of the ultimate cause of yellow fever, and that for inability to ascend up to the very throne of the Infinite, in the researches and comprehensions of our finite minds, they have failed to establish their claims as successful investigators into the causations of disease. The sentence of death has passed upon all men, this fate is inevitable; it is given to man only to arrest and stay for a time the arrows of death, and that he has this power we most firmly believe, and with this power we may rest content. If we can discover the secondary causes concerned in the development and propagation of disease, we possess the knowledge requisite to protect our race from the effects of the specific cause which has been licensed to remind us of the uncertainty of the tenure with which we hold our habitations upon earth.

A mathematical demonstration of the correctness of the deductions of the commission is not to be looked for. We cannot measure with a rule and line the capacity and power of all the elements concerned in the mysterious phenomena of life and death, any more than we can gauge, and comprehend all the wonders of the physical world. It will be time soon enough for medicine to blush for incompetency, over her inability to discover to the naked eye all the hidden mysteries wrapped up in this fleshy mantle which clothes us with mortality, when philosophy shall give us things, for names, and when we shall be able to see, feel, and gain knowledge of by all our senses, of those principles of which they talk so learnedly, but know nothing of except through their modes of manifestation to the appreciation of the human mind. Philosophy talks learnedly of gravitation, chemical affinity, electricity, but who has seen either? what are these things or elements? do we know anything of them except that they are the manifestations of some unseen power exerting itself in a prescribed form for the accomplishment of some end concerned in the economy of the world's government? The world uncomplaining, sits down in its ignorance of these things, thankfully receives whatever of development is made in the deduction of their laws of action, and cheerfully accords honors to the laborer for what progress he may make in his investigation.

Society should demand no more of medicine. The value of the labors of this commission, and the correctness of the deductions drawn from the facts and data which they have collected, should be judged by the ordinary rules applicable to other species of evidence gathered in the elucidation of an obscure subject. Progress in medicine, above all the other sciences, is slow. Truths are slowly evolved. Patient research alone will accomplish anything reliable. The light of to-day may be darkness in the brightness of to-morrow's sun; but he who has patiently watched for the coming on of the full tide splendor, deserves honor for his faithfulness at his post, and should not be forgotten in the day of the settlement of accounts.

Admitting that future revelations should entirely invalidate the correctness of the deductions drawn by the commission from the evidence before it, it is difficult to conceive how that can mitigate against the justness of the demands for payment of the services it has rendered. The members of that body, individually and collectively, have brought the full energies of their mind to the task imposed upon them, and the results of their labors are before the public.

We have unconsciously extended this subject to this length; we had designed making it only the text for illustrating the general want of appreciation upon the part of the public of all the duties which medicine is called upon to render in its behalf, and the perfect indifference with which it listens to every demand for a proper reward for these services. The claims upon medical men are daily, too, becoming greater and more burthensome. Health is now not the only subject over which they are placed as guardians, but the physician is daily becoming more and more necessary in the protection of the community against criminal violence, and the medical man is made to assume a place in the detective police of the land. The daily records of our criminal courts prove how necessary his opinions and knowledge are felt to be in the proper execution of the laws. And for all the services he thus renders, let us ask what is his reward?

But we have exhausted our limits, and perhaps we need no farther remarks to elucidate our subject, after the striking example we have given in this article.

---

*Homœopathic Principles Unfolded.*—A homœopathist, (so called) practicing in Niagara county, N. Y., having the charge of a case of sickness of aggravated form, by which the sympathising neighbors and friends became alarmed, and were assiduous and frequent in their attentions, and which case at a certain stage became fully developed *small-pox*, and thus exposed a large circle to that baneful malady, *ostensibly* assigned as ther eason that he did not sooner raise the warning voice, and so avoid the spread of the contagion, "that it was a principle of their practice, not to make known the true character of *small-pox* until the cutaneous eruption was fully manifest." *

*Ourselves.*—We are endeavoring to clear up our obligations to correspondents. It will be noticed that we have again a purely original number. The publication of Prof. Hamilton's valuable reports on fractures will be completed next month. We have, in lieu of them, made arrangements for a series of hospital reports, which bid fair to be eminently practical and valuable.

It is proper to add that we feel pleased and satisfied with the present position of the Journal, and have placed ourselves in shape to give a closer personal attention to it than heretofore. With the June number we shall enter upon our "teens," that number beginning our thirteenth volume. We hope to see it through its thirtieth before we resign the editorial chair, and with our present admirable arrangements for the business department, we expect a continued prosperity.

